# A randomized safety and pharmacokinetic trial of daily tenofovir 1% gel in term and near-term pregnancy

**DOI:** 10.7448/IAS.19.1.20990

**Published:** 2016-09-21

**Authors:** Richard H Beigi, Lisa M Noguchi, Elizabeth Montgomery, Joseph Biggio, Craig W Hendrix, Mark A Marzinke, James Y Dai, Jason Pan, Ratiya Kunjara Na Ayudhya, Jill L Schwartz, Karen Isaacs, Jeanna M Piper, D Heather Watts

**Affiliations:** 1 Department of Obstetrics, Gynecology and Reproductive Sciences, Magee-Womens Hospital of UPMC, Pittsburgh, PA, USA; 2 Microbicide Trials Network, Magee-Womens Research Institute, Pittsburgh, PA, USA; 3 Department of Medicine, Clinical Pharmacology, The Johns Hopkins University, Baltimore, MD, USA; 4 RTI International, San Francisco, CA, USA; 5 Department of Obstetrics and Gynecology, University of Alabama at Birmingham, Birmingham, AL, USA; 6 Statistical Center for HIV/AIDS Research and Prevention, Seattle, WA, USA; 7 Department of Obstetrics and Gynecology, CONRAD/Eastern Virginia Medical School, Arlington, VA, USA; 8 Pharmetrics, Chapel Hill, NC, USA; 9 Division of AIDS, National Institute of Allergy and Infectious Diseases, Bethesda, MD, USA; 10 Office of the Global AIDS Coordinator and Health Diplomacy, US Department of State, Washington, DC, USA

**Keywords:** HIV, pregnancy, prevention, pharmacokinetics, safety, tenofovir

## Abstract

**Introduction:**

Vaginal tenofovir (TFV) 1% gel may reduce incident HIV-1 and herpes simplex virus 2 infection. Pregnancy may increase risk of HIV acquisition, and incident HIV in pregnancy potentiates perinatal HIV transmission. Our objective was to investigate the safety and pharmacokinetics of seven days of TFV 1% vaginal gel in term and near-term pregnancy.

**Methods:**

Ninety-eight healthy pregnant women, stratified to a term cohort followed by a near-term cohort, were enrolled into a 2:1 randomized, double-blinded, placebo-controlled trial. Women received TFV or placebo gel for seven consecutive days with pharmacokinetic sampling on days 0 and 6. Maternal and cord blood were collected at delivery. Primary end points included laboratory and genital adverse events, adverse pregnancy and neonatal outcomes, and maternal TFV levels.

**Results:**

Most adverse events were grade 1 and none of the grade 3 or 4 adverse events were related to study product. There was no significant difference in safety end points between the two pregnancy cohorts (*p=*0.18); therefore, their data were combined. Primary safety end point rates were similar for mothers randomized to the TFV gel vs placebo arm (72.7 and 68.8%, *p=*0.81). The same was true for newborns in the TFV gel vs placebo arms (4.5% vs 6.3%, *p=*0.66). All women randomized to TFV had quantifiable serum levels within eight hours of dosing, with low overall median (interquartile range) day 0 and day 6 peak values (3.8 (2.0 to 7.0) and 5.8 (2.6 to 9.4) ng/mL, respectively).

**Conclusions:**

Daily TFV 1% vaginal gel use in term and near-term pregnancy appears to be safe and produces low serum drug levels.

## Introduction

HIV infection remains a significant global health problem, with women facing a disproportionate risk of acquisition [1]. Despite progress in HIV prevention, an estimated 2.1 million new cases of HIV occurred in 2013 [1]. Importantly, the efficacy of oral antiretroviral pre-exposure prophylaxis (PrEP) to prevent HIV infection has been demonstrated and the Centers for Disease Control and Prevention and the World Health Organization have issued relevant clinical practice guidelines for use of Truvada^®^ in this regard [2–6].

However, data are mixed on the effectiveness of topical PrEP with tenofovir (TFV) 1% vaginal gel [7,8]. The Centre for the AIDS Programme of Research in South Africa (CAPRISA) 004 trial demonstrated a 39% reduction in incident HIV-1 infections with coitally dependent TFV gel [7]. By contrast, in the Microbicide Trials Network (MTN) Vaginal and Oral Interventions to Control the Epidemic (VOICE) trial among 5029 women in sub-Saharan Africa, daily TFV 1% vaginal gel did not demonstrate benefit in HIV prevention [8]. Similarly, the Follow-on African Consortium for Tenofovir Studies 001 study reported no effectiveness of TFV 1% gel for HIV prevention in women [9]. CAPRISA 004 noted a 55% reduction in incident herpes simplex virus 2 (HSV-2) infections among TFV gel recipients, and similar protection against incident HSV-2 was noted in the VOICE study [10,11]. Given these mixed data, the future of TFV gel for HIV prevention is currently unclear; however, other delivery systems are under study for topical delivery of intravaginal TFV.

Pregnant women are a high priority population for HIV prevention. Observational data suggest that pregnancy may increase risk of HIV acquisition, and pregnant women with untreated HIV infection appear more likely (hazard ratio 2.47, 95% CI: 1.26 to 4.85) to transmit HIV to their uninfected male sexual partners when compared to non-pregnant women [12,13]. In addition, multiple investigations demonstrate that acquisition of HIV during pregnancy increases the risk for mother-to-child transmission of HIV 3- to 15-fold [14,15].

We previously reported results from a phase 1 study of single-dose TFV 1% vaginal gel among 16 pregnant women at term [16]. The favourable safety and pharmacokinetic (PK) profile in that study supported further investigation among a larger group of pregnant women using daily dosing. The primary objectives of the current investigation were to assess the safety and PK of seven consecutive daily doses of TFV 1% vaginal gel among term and near-term pregnant women in the United States. Targeting enrollees first at advanced gestational ages was a deliberately chosen pathway as part of a larger plan for sequential, backwards enrolment of pregnant women at progressively earlier gestational ages, based on favourable findings from interim safety analyses.

## Methods

An expanded phase 1 investigation of the safety and PK of seven consecutive days of TFV 1% vaginal gel among healthy term and near-term pregnant women was conducted by the MTN (MTN-008). MTN-008 was a randomized, double-blinded, placebo-controlled trial of TFV 1% vaginal gel vs hydroxyethyl cellulose (HEC) “placebo” gel (clinicaltrials.gov NCT01136759) [17]. Two study sites, Magee-Womens Hospital of the University of Pittsburgh Medical Center and the University of Alabama at Birmingham, enrolled study participants from April 2011 through September 2013. All study materials were reviewed and approved by institutional review boards at both sites. All participants underwent a thorough informed consent process. Study gel and applicators were provided by CONRAD (Arlington, VA, USA).

Healthy HIV-uninfected women (based on previous testing, as well as HIV enzyme immunoassay testing done at screening) with a singleton pregnancy were recruited from antenatal clinics. The first cohort of women was enrolled at term (37 to 39 weeks gestation, target *N=*45). After completion of study activities for all women in the term cohort, an independent review of safety and outcome data verified that no findings precluded enrolment of a subsequent cohort of near-term pregnant women. Women at 34 0/7 to 36 6/7 completed weeks of gestation (target *N=*45) were then enrolled and underwent study procedures identical to the term cohort.

Participants were randomized to active versus placebo gel in a 2:1 ratio for both pregnancy cohorts at both study sites. The targeted sample size for each pregnancy cohort allowed detection of a large difference in the primary safety end point. For an adverse event with a rate of 10% in the placebo arm, our sample size provided 80% power to detect a significantly different rate of 27% in the active gel arm. Specifically, we had 80% power to detect a statistically significant 17% difference (i.e. 10% vs 27%) in the active gel arm. The randomization was performed by an independent statistician using block sizes of six and three.

Inclusion and exclusion criteria were the same in both gestational age cohorts. Women with hypertension, diabetes, other known maternal disease, placental or foetal abnormalities, or history of preterm birth were ineligible. All participants underwent screening for HIV, other sexually transmitted infections and laboratory evidence of renal and liver dysfunction prior to enrolment. On the day of enrolment, physical and pelvic examinations were performed, maternal blood was collected pre-dose, a normal foetal heart rate was detected and noted via foetal doppler, and a single randomly assigned vaginal dose of TFV 1% gel (40 mg TFV) or HEC placebo was administered by blinded study personnel. Women underwent subsequent blood collections to determine serum TFV concentration at one, two, four, six, and eight hours after gel administration. The same in-clinic study procedures took place on day 6. Women were dispensed applicators pre-filled with study product and were directed to insert study product at home once daily for five consecutive days (days 1 to 5).

Women had baseline and day 6 laboratory and genital tract assessments. In addition, women were contacted by phone on days 1 and 3 of gel use, as well as day 14 (one week after completing gel use), to query for additional adverse events (AEs) or questions surrounding gel use. Study staff attended deliveries to collect maternal and cord blood samples for serum TFV concentrations as well as data on peripartum AEs. Participants were contacted again at two weeks after delivery to collect any additional maternal or neonatal AEs.

Primary maternal safety end points included grade 2 or higher AEs for laboratory end points (liver and kidney function), genital tract signs/symptoms and specific pregnancy outcomes as defined by the Female Genital Grading Table for Use in Microbicide Studies [18]. The relatedness of AEs to study product was determined by site primary investigators. Primary infant end points included admission to the neonatal intensive care unit (NICU) for greater than 24 hours or the diagnosis of neonatal sepsis in the first seven days of life. Secondary end points included assessments of adherence to daily use of TFV 1% gel for seven days and its acceptability among pregnant women. The results of these adherence and acceptability secondary end points will be reported in separate publications.

### Tenofovir assays

Blood was centrifuged within eight hours of collection and the serum was frozen at –70°C. TFV concentrations were measured via liquid chromatographic–tandem mass spectrometric (LC-MS/MS) analysis, with a lower limit of quantitation of 0.31 ng/mL [19]. The LC-MS/MS method was validated in accordance with recommendations specified in the Food and Drug Administration's *Guidance for Industry: Bioanalytical Method Validation* guidelines [20]. PK parameters were estimated including peak concentration (C_max_), time to C_max_ (T_max_) and area under the concentration versus time curve from zero to eight hours after dosing (AUC_0-8_). PK parameters were compared within individuals between day 0 and day 6 and between cohorts using paired and unpaired Wilcoxon rank sum tests, respectively, using exact tests with statistical significance at *p<*0.05.

### Analyses of study outcomes

Data analysis included all participants who were randomized and received study products. Descriptive statistics were used to summarize the characteristics of the study population, such as age and race. Adverse events were evaluated for both pregnancy cohorts, initially stratified by cohort, then combined due to lack of significant differences noted between the two cohorts (*p=*0.18 for difference between cohorts). The number and the percentage of AEs were tabulated by severity, category and relationship to study product. The proportion of participants having at least one AE event was compared between arms using Fisher's exact test. Non-compartmental PK was used to estimate pre-dose concentration, C_max_, T_max_ and AUC_0-8_, which were summarized using descriptive statistics. These values were also compared to non-pregnant historical controls to assess for differences between pregnant and non-pregnant women in PK parameters [19].

## Results

**Figure 1:**
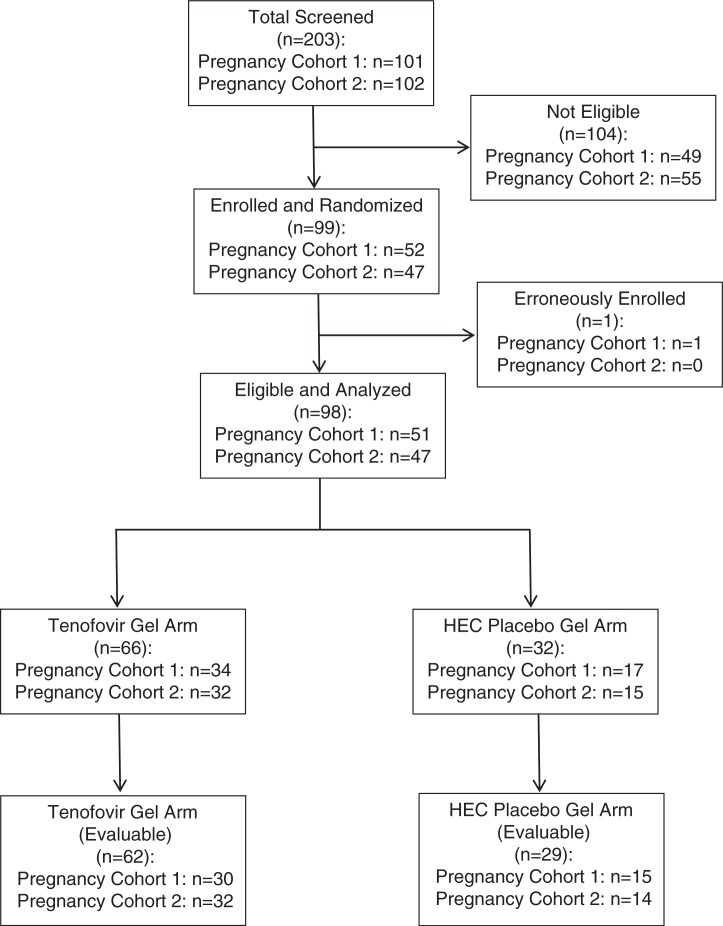
Participant flow diagram.

A total of 203 women were screened and 99 women were enrolled with the goal of having at least 90 evaluable women (assuming roughly 10% would be non-evaluable) women receiving at least four doses of study product and completing the day 6 visit (Figure 1). Of these 99, 98 received study gel and contributed to the safety and PK data for analysis (51 term, 47 near-term). None of the participants were lost to follow-up. No abnormalities in foetal heart rate or complaints related to foetal well-being were detected during the dosing interval. Participant demographic characteristics are shown in Table 1.

**Table 1:** Demographic characteristics of participants, by cohort

	Pregnancy cohort group 1 (term) mothers (*N=*51)	Pregnancy cohort group 2 (near-term) mothers (*N=*47)
Age (years)		
Mean (SD)	24.2 (4.5)	23.5 (4.7)
Median, min to max	23.0, 18.0 to 40.0	22.0, 18.0 to 38.0
Race		
Black or African American	37 (73%)	40 (85%)
White	10 (20%)	4 (9%)
Mixed race^a^	4 (7%)	3 (6%)

a Cohort group 1: Two participants were black or African American/white, one participant was Asian/white, one participant was Latina and one participant was American Indian or Alaskan Native/black or African American/white. Cohort group 2: Three participants were black or African American/white.

Table 2 describes primary maternal end points by study arm for both gestational age cohorts combined. No significant differences were noted between study arms in specific maternal adverse events, total maternal events or infant primary outcomes. Any maternal end point (grade 2 or higher AE), regardless of treatment relatedness, was seen in 48 (73%) of 66 women in the TFV gel group and 22 (69%) of 32 women in the placebo group (*p=*0.81). Five infants had a neonatal primary end point and required NICU care for ≥24 hours, 3 (4%) of 66 in the TFV gel group and 2 (6%) of 32 in the placebo group (*p=*0.66), including respiratory distress syndrome (*n=*2), neonatal hypoglycaemia (*n=*2) and pneumonia (*n=*1). All infants recovered and were doing well at time of discharge.

**Table 2:** Primary maternal end points during the dosing interval and at delivery^a^^b^

	Tenofovir gel (*N=*66)	HEC placebo gel (*N=*32)	*p*
Specific pregnancy complications			
Post-partum haemorrhage	13 (19.7%)	4 (12.5%)	0.57
Post-partum endometritis	1 (1.5%)	0	1.00
Chorioamnionitis	1 (1.5%)	2 (6.3%)	0.25
Third trimester bleeding	1 (1.5%)	1 (3.1%)	0.55
Term premature rupture of membranes	15 (22.7%)	6 (18.8%)	0.80
Spontaneous preterm delivery	2 (3.0%)	3 (9.4%)	0.33
Genital and pelvic signs/symptoms			
Pain (vulvar, vaginal and/or pelvic)	25 (37.9%)	14 (43.8%)	0.66
Lesions (vulvar, vaginal and/or cervical)	2 (3.0%)	1 (3.1%)	1.00
Vulvovaginitis	1 (1.5%)	0	1.00

a There were no events of preterm premature rupture of the membranes, liver or kidney function laboratory abnormalities, cervicitis or vulvar/vaginal rashes, tenderness, itching, edema, erythema, dryness or dysuria

b None were considered treatment-related. HEC, hydroxyethyl cellulose.

A total of 377 maternal and 63 infant AEs were noted from both pregnancy cohorts during study follow-up (Table 3). Proportions of maternal and neonatal AEs by gestational age cohort were statistically comparable and were combined for further analyses. No maternal or infant deaths occurred. Most maternal AEs were grade 1 or 2 and were deemed unrelated to the study product (93%). The grade 1 AEs considered related to the study product were predominantly self-limited genital tract signs and symptoms (e.g. vaginal discharge and/or discomfort), and the two moderate AEs considered related were self-limited diarrhoea and pruritus. None of the grade 3 to 4 AEs were related to the study product (all were related to pregnancy, delivery and the post-partum period). Of the 63 infant AEs, most were grade 1 or 2 events (87%) and none were deemed related to study product.

**Table 3:** Maternal and infant adverse events during study participation (by pregnancy cohort)^a^

Maternal (term)	Tenofovir gel arm (*N=*34)	HEC placebo gel arm (*N=*17)	p*	All arms (*N=*51)
Related^b^	5 (6)	4 (5)	0.46	9 (11)
Not related	26 (122)	13 (54)	1.00	39 (176)
Total	31 (128)	17 (59)	0.54	48 (187)
**Maternal (near term)**	**Tenofovir gel arm (*****N=*****32)**	**HEC placebo gel arm (*****N=*****15)**	p*	**All arms (*****N=*****47)**
Related^c^	10 (13)	2 (3)	0.29	12 (16)
Not related	21 (126)	13 (48)	0.17	34 (174)
Total	31 (139)	15 (51)	1.00	46 (190)
**Infant (term)**	**Tenofovir gel arm (*****N=*****34)**	**HEC placebo gel arm (*****N=*****17)**	p*	**All arms (*****N=*****51)**
Related	0 (0)	0 (0)	–	0 (0)
Not related	13 (21)	8 (11)	0.56	21 (32)
Total	13 (21)	8 (11)	0.56	21 (32)
**Infant (near term)**	**Tenofovir gel arm (*****N=*****32)**	**HEC placebo gel arm (*****N=*****15)**	p*	**All arms (*****N=*****47)**
Related	0 (0)	0 (0)	–	0 (0)
Not related	12 (22)	7 (9)	0.75	19 (31)
Total	12 (22)	7 (9)	0.75	19 (31)

HEC, hydroxyethyl cellulose.

a Data are presented as number of participants with adverse events (actual number of adverse events are in parentheses)

b All adverse events that were considered as related to study product in the maternal term cohort were mild

c Among 16 adverse events that were considered as related to study product in the maternal near-term cohort, only two were moderate and both were from the tenofovir gel arm (diarrhoea and generalized pruritis).

* *p*-values were based on Fisher's exact test of comparison of the number of participants experiencing adverse events between arms.

**Figure 2:**
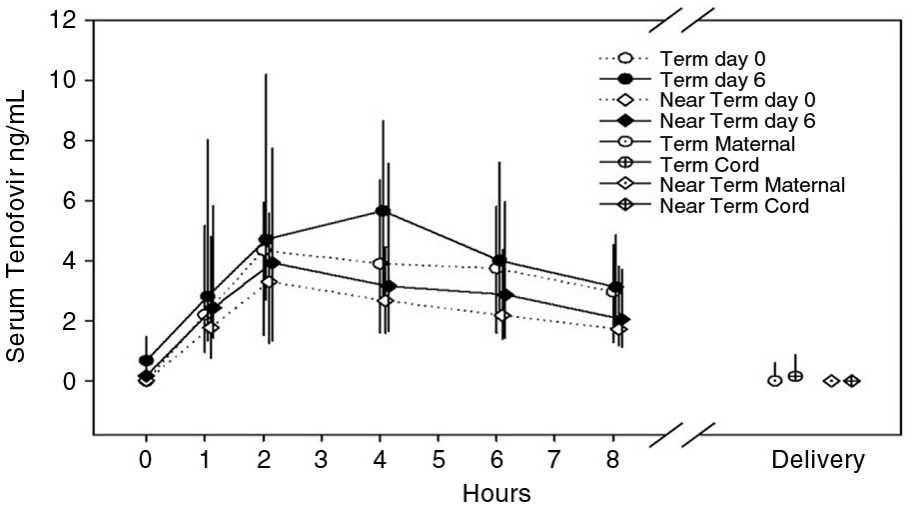
Concentration vs time curve for tenofovir gel, day 0 and day 6. (*“Term maternal” and “Near term maternal” refer to levels in mother at the time of delivery.)

The term and near-term cohorts were not different in terms of serum concentration vs time profiles (Figure 2) or C_max_ and AUC_0-8_ (*p>*0.05) for TFV. Accordingly, these cohorts were pooled for subsequent analyses. All 66 women randomized to TFV gel had quantifiable concentrations (>0.31 ng/mL) noted during the eight hours after dosing. Fifty-five percent of women randomized to daily TFV gel had quantifiable levels at the pre-dose blood draw on day 6 indicating potential accumulation of drug throughout the week based on previously established TFV plasma kinetics. C_max_ and AUC_0-8_ for all participants were greater on day 6 compared to day 0 (*p=*0.004 and *p=*0.002, respectively). Full PK data, as well as historical references to non-pregnant participants exposed to TFV gel for comparison, are shown in Table 4.

**Table 4:** Pharmacokinetic parameters on day 0, day 6 and at delivery among women randomized to tenofovir gel (*N=*66)

Time point	Tenofovir gel arm^a^ (*N=*66)	Non-pregnant comparator^b^
Day 0		
C_max_ (ng/mL)	3.8 (2.0 to 7.0)	4.0 (1.5 to 9.1)
T_max_ (hours)	2.0 (2.0 to 4.0)	4.0 (2.0 to 6.0)
AUC (ng^a^h/mL)	7.9 (4.0 to 16.8)	
Day 6		
Pre-dose (ng/mL)	0.5 (BLQ to 1.0)	1.8 (0.5 to 3.5)*
C_max_ (ng/mL)	5.8 (2.6 to 9.4)**	3.9 (2.2 to 7.9)
T_max_ (hours)	2.0 (2.0 to 4.0)	2.1 (1.9 to 4.6)
AUC (ng^a^h/mL)	12.2 (6.6 to 19.2)**	10.1 (5.2 to 19.1)
Delivery		
Maternal (ng/mL)	BLQ (BLQ to BLQ)	
Cord blood (ng/mL)	BLQ (BLQ to 0.36)	

MTN, microbicide trials network; AUC, area under the curve; BLQ, below limits of quantification; IQR, interquartile range.

a All values are median (IQR)

b data available [17,26].

* *p*<0.05 compared to MTN-008 day 6 cohort

** *p*<0.05 compared to day 0 dosing.

The median interval between last dose of study drug and delivery was 14 days, with a range of 0 to 41 days. Thirteen percent of women had quantifiable TFV in blood at delivery (maximum of 25 days after last gel use) at very low levels (<1.18 ng/mL) compared to plasma concentrations after oral dosing. Twenty-three percent of infants had quantifiable TFV in cord blood, also at very low levels (<2.17 ng/mL). The longest interval of quantifiable TFV in cord blood after last day of drug exposure was 25 days in the same mother–infant dyad (1.44 ng/mL). Despite this atypical finding, neither TFV detection nor concentration at delivery (maternal or cord blood) was statistically related to time from last dose.

## Discussion

This investigation among term and near-term pregnant women found a reassuring safety profile with daily TFV gel use and no obvious evidence of untoward pregnancy outcomes. PK findings suggest that absorption among term and near-term pregnant women using the gel daily is similar to non-pregnant women and produces low overall systemic exposure for the mother and foetus. While TFV was quantifiable in roughly a quarter of the newborns at the time of delivery, concentrations were low and not associated with detectable adverse outcomes. These data suggest that TFV gel use among pregnant women at or near term appears to be safe and would be unlikely to pose a significant risk to mothers or their newborns.

Our previous work failed to detect any safety signal and noted low exposure and limited drug transfer via the placenta of single-dose TFV gel use in 16 term pregnant women (16). The current study was the next step to investigate the safety and PK of this candidate HIV prevention drug with more extended exposure (albeit modest at seven days) among a larger sample size. The stepwise approach of enrolling pregnant women at term, carrying out an interim safety analysis and subsequently enrolling near-term women was a deliberately cautious strategy [21]. Consistent with this approach, the next planned investigation would have entailed daily dosing of pregnant women for 28 consecutive days at progressively earlier points in pregnancy, going back until the end of the first trimester, with interim safety analyses prior to embarking on each successive earlier gestational age regimen. This approach was supported by reassuring safety data available at the time of protocol development on oral TFV use in pregnancy [22], single-dose TFV gel in pregnancy [16], as well as from numerous studies of TFV gel use among non-pregnant women [19,23,24]. Since the design and performance of this study, newer data suggest that longer-term (weeks) exposure *in utero* to TFV via maternal oral dosing for HIV treatment in pregnancy may have effects on neonatal growth and bone density [25,26]. The relevance of these more recent findings among infants exposed to significantly higher drug levels via oral dosing (roughly 50- to 100-fold higher compared to vaginal dosing) for significantly longer periods of time is unclear. Data from a separate ongoing HIV prevention agent exposure registry (MTN-016) could permit investigation into potential impact on growth parameters in the first year of life among infants exposed to TFV gel [27].

The future of TFV gel for HIV prevention is currently uncertain. Accumulating and intriguing data suggesting that TFV gel has efficacy in prevention of new HSV-2 infections is relevant to women at risk for HIV and other sexually transmitted infections [7,10,11]. Incident HSV-2 infections particularly in the late second or third trimester carry a significant risk of neonatal HSV infection with associated severe health consequences [28]. A novel approach to prevention of incident HSV-2 in pregnancy could hold considerable potential for curbing HSV-2-related neonatal morbidity and mortality.

Despite the relatively small size, the lack of an obvious safety signal provides some reassurance about future investigations and subsequent potential use of this product in pregnancy. The combination in this study of the non-worrisome pregnancy outcomes and the novel study design warrants attention despite the unclear future of TFV gel [8,9]. Although this was a relatively small study with limited power to detect either rare adverse outcomes or other outcomes occurring at modest rates, use of a control arm helps to suggest that the AEs noted were related to the pregnancy itself and not TFV gel. Enrollees in both arms had modest and equivalent rates of untoward pregnancy and neonatal outcomes that fell within expected population background rates [29].

Our PK findings indicate that term and late preterm pregnant women have the same low peak drug concentrations and systemic exposure as non-pregnant women when using the same analytical methods [19]. Specifically, MTN-008 pregnant women were not different in terms of C_max_, T_max_ and AUC_0-8_ (Table 4) when compared to steady-state values in a non-pregnant historical cohort of women taking daily TFV 1% gel [19]. Additional reports in non-pregnant women receiving TFV 1% gel have noted peak TFV concentrations that largely resemble our results [23,30]. Because the concentration–time profile after an observed initial dose is consistent between pregnant and non-pregnant women, the statistically significant difference in pre-dose (day 6) TFV concentrations may be due to a small adherence difference prior to sampling or to small PK differences between non-randomized populations. The magnitude of the difference is small compared to post-dose concentrations and does not affect the key finding of highly concordant TFV concentrations between pregnant and non-pregnant populations with observed dosing. This understanding provides confidence in our interpretation of the reassuring safety findings, albeit in a relatively small population.

This study has several strengths, including (1) novel pregnancy study design, (2) partially directly observed dosing on days 0 and 6 and (3) inclusion of a placebo gel arm, permitting a more robust comparison. However, the sample size and associated low power of the study limit the ability to draw definite conclusions regarding safety. While women enrolled as early as 34 weeks, this precludes an understanding of safety earlier in pregnancy. Several other limitations should also be noted. By design, product use occurred among a preselected group of healthy pregnant women, challenging generalizability. While vaginal tissue concentrations of tenofovir diphosphate (TFV-DP), unlike serum TFV concentrations, may not have fully achieved steady-state concentrations by six days in our study, we believe these women had very nearly achieved steady state (>85%) based on prior reports of TFV-DP tissue half-life, between two and three days in peripheral blood mononuclear cell (PBMC), vaginal tissue and vaginal tissue cells [31]. Based on the colon and vaginal tissue kinetics of TFV-DP, the concentrations reported in our previous single-dose study of term infants and their mothers (MTN-002) likely represent concentrations less than half of steady-state concentrations. Our current report goes further in working to establish a safety profile for longer dosing periods and at earlier stages of pregnancy.

## Conclusions

TFV gel use in term and near-term pregnancy appears to be safe and produces low serum drug levels. This study is an important sequential step in understanding the safety and PK of TFV 1% gel in pregnancy. Should an indication for HSV-2 prevention be sought for TFV gel or other topical formulation, these data can help form a foundation for future investigations to assure that pregnant women can use it safely at or near term. The approach used herein will be informative for future pharmaceutical products seeking licensure for use by reproductive age women.
